# Peritoneal Response to Abdominal Surgery: The Role of Equine Abdominal Adhesions and Current Prophylactic Strategies

**DOI:** 10.1155/2014/279730

**Published:** 2014-01-20

**Authors:** Juliana de Moura Alonso, Ana Liz Garcia Alves, Marcos Jun Watanabe, Celso Antonio Rodrigues, Carlos Alberto Hussni

**Affiliations:** School of Veterinary Medicine and Animal Science, UNESP, University Estadual Paulista, Botucatu, São Paulo, Brazil

## Abstract

Intra-abdominal adhesions constitute a significant clinical and surgical problem that can lead to complications such as pain and bowel occlusion or subocclusion. These adhesions are frustrating and potentially fatal, representing a major postoperative complication in abdominal surgery. It is estimated that 32% of horses undergoing laparotomy will present clinical symptoms due to adhesions, but the true prevalence is not known because a large proportion of animals with postoperative recurrent colics are medically treated or submitted to euthanasia without necropsy. Adhesions are highly cellular, vascularized, dynamic structures that are influenced by complex signaling mechanisms. Understanding their pathogenesis could assist in applying better therapeutic strategies and in developing more effective antiadhesion products. Currently, there are no definitive strategies that prevent adhesion formation, and it is difficult to interpret the results of existing studies due to nonstandardization of an induction model and evaluation of their severity. The best clinical results have been obtained from using minimally traumatic surgical techniques, anti-inflammatory agents, antimicrobials, anticoagulants, and mechanical separation of serosal surfaces by viscous intraperitoneal solutions or physical barriers. This paper aims to review adhesion formation pathogenesis, guide the understanding of major products and drugs used to inhibit adhesion formation, and address their effectiveness in the equine species.

## 1. Introduction

Intra-abdominal adhesions in humans and animals constitute a significant clinical and surgical problem that can lead to complications such as pain, infertility, and bowel occlusion or subocclusion [1–4]. These adhesions also have high economic impacts due to surgical and hospital expenses [2, 5]. Adhesion formation is the most frequent cause of postoperative colic and the second most common cause of repeated celiotomy [6–8]. Therefore, they are an object of frustration for veterinary and human surgeons, which has stimulated research into products and methods to prevent their formation [9].

Although the trigger mechanisms of adhesion formation remain unclear, the possible causes are ischemia, surgical trauma, inflammation, hemorrhage, thermal or chemical injury, genetic predisposition, and reactions to foreign bodies [1, 5].

## 2. Review

### 2.1. Adhesion Formation Mechanism

The peritoneal membrane is embryologically derived from mesothelial cells. It is anchored in a basement membrane of submesothelial layer, the extracellular matrix (ECM). ECM consists of collagen, glycoproteins, glycosaminoglycans, and proteoglycans. Mesothelial cells are sensitive to minimal trauma and possess the capacity to secrete interleukins (IL)-1 and IL-6, tumor necrosis factor (TNF)-*α*, and transforming growth factor (TGF)-*β*. These cells also contribute to the fibrinolytic process through the secretion of tissue plasminogen activator (tPA) and inhibitors of tissue plasminogen activator (PAI) [1].

After tissue injury, the vascular response is a brief vasoconstriction followed by histamine and prostaglandin E2 (PGE2) release, which results in local vasodilatation and cell influx, making the environment inflammatory and attracting procoagulant factors through the local vasculature and peritoneal fluid. Platelets in this inflammatory exudate adhere to the wound bed and suffer degranulation of their alpha corpuscles, which release platelet-derived growth factor (PDGF) and TGF-*β*, and dense corpuscles, which release epinephrine and serotonin. These events contribute to prostaglandin and leukotriene release [3, 10].

Local release of cytokines stimulates cell migration to the wound bed, while platelet aggregation contributes to coagulation cascade activation and initial fibrin clot formation, initiating the clotting process. Fibrin deposition acts as a temporary matrix of signaling molecules and inflammatory cells, and as a temporary bridge between tissues [10].

Coagulation and inflammation are closely related. The coagulation system is divided into two pathways that converge to a common pathway. This process results in thrombin activation and fibrin formation. The intrinsic pathway is composed of several proteins activated by Hageman's factor (XII) in the presence of basement membrane damage and collagen exposure, which results in coagulation stimulation through the activation of several proteins, among them prothrombin (factor II) and the precursor of thrombin (factor IIa). Thrombin is the major link between inflammation and coagulation, as this enzyme is responsible for cleavage of soluble circulating fibrinogen in insoluble fibrin clots [11].

Factor XII stimulates blood clot formation at the same time that the fibrinolytic system is activated. By cleaving fibrin, fibrinolytic system is the main modulator of adhesion formation. Tissue plasminogen activator (secreted by mesothelial cells, leukocytes, and tissue) cleaves plasminogen to generate plasmin, a protease that acts in fibrin lysis, transforming fibrin into fibrin degradation products (FDPs). Fibrinolysis is regulated by type 1 and type 2 inhibitors of tissue plasminogen activator (PAI-1 and PAI-2, resp.), which are stimulated in the presence of trauma, infection, or endotoxin [11–14].

Plasmin also acts in complement system cascade activation, stimulating kinins that increase vascular permeability, allowing the influx of more inflammatory cells into the abdominal cavity after surgical trauma [11]. In the first 12–24 hours, the recruited cells are polymorphonuclear (PMN) cells, predominantly neutrophils. As the inflammatory process evolves, after 24 hours, the exudate becomes dominated by macrophages, cells that act in debridement and phagocytosis of pathogens and in directing the healing process through the release of cyclooxygenase, lipoxygenase, plasminogen activators, inhibitors, collagenase, IL-1, IL-6, TNF, leukotrienes, and prostaglandins, along with acting in the mesothelial cells, endothelial cells and fibroblasts. On approximately the third day, mesothelial cells and fibroblasts start to cover the area and macrophages infiltrate in the tissue. The area of the lesion regresses, and the mesothelial layer is reestablished and covers the lesion between the seventh and the tenth days. [3, 10, 15, 16].

ECM deposition occurs simultaneously with cell migration and proliferation and is mediated by fibroblasts and possibly driven by macrophages. ECM formation in normal healing or adhesion formation results from the interaction between growth factors (TGF-*β*, EGF, IGF, FGF and VEGF) and bioactive molecules. TGF-*β* is locally produced and closely associated with neutrophil, T cell, monocyte and fibroblast chemotaxis, and with the production of various ECM protein constituents, such as fibronectin, glycosaminoglycans, and collagen [10].

In cases where peritoneal healing occurs abnormally, mesothelial cells, fibroblasts, and peritoneal macrophages signal excessive deposition of ECM. Fibroblasts and myofibroblasts secrete large amounts of ECM components, such as fibronectin, hyaluronic acid, glycosaminoglycans and proteoglycans, establishing a fibrous bridge formation between tissues. Vascularization, collagen deposition, and slow resorption of water strengthen the bridge formed, providing the necessary tension, which results in proper adherence between tissues [5, 10].

Fibrinolysis varies between species [17]. In horses, few studies have assessed fibrinolytic activity in peritoneal fluid. Compared to humans, horses have elevated levels of proteins that inhibit coagulation (antithrombin and protein C) and fibrinolytic components (plasminogen, fibrinogen, and FDPs) after abdominal surgery [14, 18].

If local fibrinolysis is adequate, fibrinous adhesions are lysed; but if it is insufficient, fibrin adhesions are perpetuated and infiltrated with fibroblasts and capillaries, leading to fibrous adhesions [14].

### 2.2. Adhesion Epidemiology

Adhesions are prevalent in people previously subjected to laparotomy; studies have reported frequencies of 67% [19], 74% [20], and 93% [21]. In horses, the incidence of postoperative adhesions with clinical manifestation is between 9 and 32% [4, 6, 14, 22–24]. However, it is impossible to determine the true prevalence of postoperative adhesions and their precise role in morbidity and postoperative mortality because most animals with postoperative recurrent colics are medically treated or euthanized without necropsy [4].

In a retrospective study, Gorvy et al. [4] reported 1014 cases of colic over six years. Of these, 99 required a second laparotomy, 32.3% (32/99) of which detected adhesions, and 84.4% of these were responsible for clinical symptoms.

In horses, adhesions are most often seen after surgeries in which the initial injury affects the small intestine (56%) compared to the large intestine (44%). Among small-intestine adhesions, 94% cause clinical symptoms, compared to 71% in the large intestine. Even in horses undergoing laparotomy due to large-bowel disease, adhesions form more frequently in the small intestine (36%) or the midline (29%) compared to the large intestine [4]. These findings allow us to infer that adhesion location may not be solely related to the initial site of injury but may also be secondary to surgical inflammation and injury distal to the primary lesion.

### 2.3. Prophylactic Strategies

Although the issue of adherence is not new, there is currently no definitive strategy to prevent its formation [5, 10]. Assessing and comparing the effectiveness of treatments are still difficult because there is no standardization of induction and grading protocols [10, 25]. Combined with appropriate surgical technique, the agents most commonly used as adjuvants in adhesion inhibition are anti-inflammatory agents, absorbable material barriers, gels or solutions, fibrinolytic agents, anticoagulants, and antioxidants [5, 9, 26, 27].

The product or material chosen must first be biocompatible and should not interfere with the normal healing process. Among all of the criteria, the most important is the effectiveness of the product [5, 28]. In horses, prophylactic strategies that have some merit are viscous solutions of high-molecular-weight polymers, such as solutions of 1% carboxymethylcellulose (CMC), hyaluronate and CMC bioabsorbable membranes, heparin, peritoneal lavage, and omentectomy [29].

The surgeon can adopt several surgical strategies to decrease the impact of adhesion formation. These strategies are logical and free of additional costs, including strict antisepsis, the use of synthetic gloves without powder, minimal surgical trauma, continuous bowel irrigation, minimal use of electrosurgery, strict hemostasis, the use of small and biocompatible sutures, and minimal tissue dissection [9, 12, 25, 30, 31]. Although surgeons widely disseminate and apply these techniques, the occurrence of adhesions persists [5].

Laparoscopy significantly decreases visceral manipulation, reducing adhesion formation/reformation in animals and humans. However, in horses, not all surgical procedures can be performed in this manner [5, 25, 31].

The effectiveness of peritoneum closure for adhesion formation is a subject of great disagreement, and though several studies indicate that peritoneum closure increases the incidence of postoperative adhesions [31–33], a systematic meta-analysis suggested that not suturing the peritoneum closed is associated with increased adhesion formation [34].

Omentectomy's influence on adhesion formation has been investigated in horses [35, 36]. Omentum removal results in decreased adhesion formation and facilitates postoperative peritoneal lavage [37, 38]. However, omentum removal is controversial because adhesions involving this structure are rarely observed [39].

Solid barriers comprise the largest category of products approved for use against adhesions formation. These products have demonstrated efficacy in reducing the incidence and severity of adhesion formation in people [5, 10, 30]. They act by forming a physical barrier between the tissues. There are many different materials in this category, but two major products are Seprafilm [40] (Genzyme Corporation) and Interceed [5, 41]. Both products are used in human patients undergoing abdominal or pelvic surgery as adjuvants for reducing adhesion formation between the abdominal wall, intestinal segments, uterus, and surrounding structures [40, 41]. Seprafilm consists of CMC and hyaluronic acid, and Interceed consists of oxidized cellulose. Both products are biodegradable and designed for a single application [10]. Seprafilm is more widely studied in human adhesion prevention. There is no consensus about these products' effectiveness, though studies in rats, mice, rabbits, and humans demonstrate that they decrease adhesion incidence, severity, and area [5, 42, 43].

In horses, among the solid barriers used, hyaluronate bioabsorbable membranes are the most prevalent [9, 44]. Eggleston and Mueller [39] report that the use of this membrane decreases morbidity and mortality in horses undergoing resection of ischemic segments.

Although there are several products that fit the classification of a liquid barrier, few is recommended for use because although they are biocompatible, they can cause tissue reactions [5]. Because of their liquid formulation, such products do not remain long enough to prevent contact between surfaces. The peritoneum is capable of absorbing large quantities of liquid in one or two days, and because adhesion formation occurs mostly between the seventh and tenth days, the fluid applied during surgery would be absorbed before a liquid barrier would prevent adhesion formation, making their use impractical [5, 10, 30].

Viscous solutions of CMC have lubricating properties, reduce handling trauma, and act as barriers to serosal surfaces. CMC demonstrate variables efficacy in rats, rabbits [5, 45] and horses [46]. Despite the low success rate in people, intraperitoneal use of 1% CMC during equine surgery apparently does not interfere with anastomosis or surgical incision healing [47–49], and it doubles the survival rate [50]. CMC solution application is recommended at the beginning of surgery and whenever necessary to keep serosal surfaces lubricated, reducing tissue damage from surgical trauma [39].

In contrast to previous theories, adhesions can be formed in any region of the abdomen after surgical trauma, with no predilection to anastomosis, enterotomy, or celiotomy incision site; thus, the use of therapeutic pan-abdominal measures may demonstrate greater efficacy in horses [4].

Although several drug strategies have been evaluated, no available product has proven efficacy [5]. The drugs most commonly used are steroidal or nonsteroidal anti-inflammatory drugs (NSAIDs), fibrinolytic agents, anticoagulants, and antioxidants [26, 27]. Broad-spectrum antibiotics, anti-inflammatory agents, and dimethylsulfoxide (DMSO) decrease inflammation and bacterial load, resulting in reduced fibrin deposition and thus decreased adhesion formation [7, 9, 39, 49]. Anticoagulant drugs are used to prevent fibrin clot formation and thus inhibit adhesion formation [5, 39].

Heparin is an acidic sulfated proteoglycan anticoagulant with varying molecular weight. Heparin catalyzes the antithrombin III inhibition reaction [51], stimulating tPA, resulting in fibrinolysis [51, 52]. There is no consensus about heparin's efficacy in adhesion formation inhibition [9], though Parker et al. [53] observed a significant decrease in adhesion formation using heparin at a dose of 40 IU/kg every 12 hours for 48 hours in horses given experimental adhesions. Young et al. [54] evaluated low-molecular-weight heparin at a dose of 66 IU/kg every 12 hours for five days in horses undergoing laparotomy, and no favorable effect was observed on postoperative complications or survival rate. In rats, heparin alone [55–57] or associated with other prophylactic therapies [58] prevents adhesion formation or reduces their severity. In people [59–61], no favorable results have been obtained. The recommended dose of heparin is still controversial. One recommendation is 20–150 IU/kg every six to 12 hours for two to five days [39, 51]. Although no intraperitoneal studies have been performed in horses, the application of 30,000 IU heparin diluted in saline solution is routinely used and has been informally described as effective [39]. After prolonged therapy with heparin, complications such as anemia, bleeding, and thrombocytopenia may occur. Associated with these complications, heparin shows a steady increase in serum concentration with treatment, so a progressive decrease in the dosage from 150 IU/kg to 125 IU/kg after three days and to 100 IU/kg after seven days of treatment is recommended [51].

The use of fibrinolytic agents such as rtPA, which binds to fibrin and activates the conversion of plasminogen to plasmin, reduces the adhesion incidence in horses, although its application is not economically feasible [39].

Oxidative stress plays an important role in the adhesion formation mechanism, mainly by suppressing mesothelial cells' fibrinolytic activity. Antioxidants, when used by the intraperitoneal route, decrease oxidative stress and increase fibrinolytic activity. *N*-Acetyl-cysteine (NAC) is an antioxidant that acts on intracellular glutathione synthesis and is believed to inhibit adhesion through active cellular mechanisms of inflammation and angiogenesis. No studies have used NAC in horses, although Pata et al. [26] used NAC intramuscularly, and Chu et al. [27] used intraperitoneal NAC in rats and observed a significant decrease in the degree of adhesions.

Prolonged postoperative ileus increases the risk of adhesions. Thus, the use of prokinetic agents may assist in the prevention of adhesions after laparotomy [23, 39].

Abdominal lavage effectively prevents adhesions by removing blood, fibrin, and inflammatory mediators [8, 9, 62]. Abdominal lavage is recommended 12, 18, 36, and 48 hours after surgery using ten liters of heated Ringer's lactate [38, 39].

In people with consistent clinical symptoms of adhesion, dietary modification, including smaller, more frequent meals, are recommended. Similarly, in horses, dietary management consists of low bulk in low volume with greater frequency [9].

### 2.4. Potential New Therapies

Currently, a huge range of antiadhesion products continue to be tested and developed in experimental models due to the absence of a wholly efficient inhibition drug or product established [39]. However, few of these new therapies present some results tested in the equine species.

The sodium hyaluronate (HA) is a naturally occurring biocompatible glycosaminoglycan consisting of repeated disaccharide units [63, 64]. Hyaluronic acid solution (0.4%) as a peritoneal lubricant is effective in inhibiting adhesion formation in horses [39]. However, its high cost has been an obstacle to use [39]. When tested in rats, HA has been shown to have limited anti-adhesion properties by itself and has been successfully used to prepare carrier films to deliver potential anti-adhesion agents to surgery sites in rat uterine horn and cecal-sidewall models of abdominal adhesions [64].

Relying on HA film use as a carrier of antiadherent products, several studies have used this material for such purpose. Cashman et al. [63] evaluated the *in vivo* efficacy of 13 compounds on adhesion inhibition and characterized the potential toxicity of the most efficacious compounds tested; all products were carried by HA, and isolated HA was the control group. HA fucoidan films reduced adhesion scores by approximately 90% compared with control films (*P* < 0.05). A total of 50% to 100% of rats were adhesion free at fucoidan film loadings of 0.33% to 33% compared with all control film animals having adhesions. No adverse effects were observed from 33% fucoidan films equivalent to approximately 30 mg fucoidan/kg body weight [64].

Fucoidans are broad molecular weight sulfated polysaccharides that are extracted from the extracellular matrix of brown macroalgae (*Laminaria japonica*), which is manufactured as a liquid concentrate (PERIDAN) [65–67]. Fucoidan possesses a variety of biological properties including antiadhesive, anticoagulative, and anti-inflammatory effects through interactions with thrombin, antithrombi III, heparin cofactor and leukocyte membrane receptors [65, 66].

In studies in laboratory animals [63] and pony foals [68], intra-peritoneal administration of fucoidan solution prior to abdominal closure demonstrated safety and efficacy in minimizing the number and severity of experimentally induced postoperative adhesions [66]. Safety has also been demonstrated in a jejunojejunostomy anastomosis model in adult horses [69].

Seeking clinical use evaluation, MacKinnon et al. [70] applied fucoidan in 33 colic patients subjected to laparotomy. Fucoidan (PERIDAN Concentrate) (50 mL) was mixed into 5 L lactate Ringer solution (LRS) or Plasmalyte solution for horses and the 5 mL into 500 mL LRS or 1 L Plasmalyte for foals. The solution was mixed and administered before abdomen closure. Although this study was not a randomized prospective clinical trial, there was some evidence of a favorable long-term outcome with regard to survival and reduction of colic particularly considering the number of neonates and the high proportion of horses with small intestinal lesions, adhesions, and that underwent repeat celiotomy [70].

Among the most recently developed therapies that include also the chitosan dextran (CD), a unique synthetic gel, its active ingredients are succinyl chitosan and dextran aldehyde [25]. CD has a variety of medical applications although its use as an adhesion prevention product has yet to be fully established [71]. Lauder et al. [25] evaluated Wistar Albino rats subjected to cecal abrasion and treated with CD gel, demonstrating that a CD gel is well tolerated as an intra-abdominal agent with a highly statistically significant reduction in adhesion formation and with no increased risk of enterotomy dehiscence when compared to control groups [71]. But, in contrast with Lauder [25, 71], Shahram et al. [78] obtained insignificant effect on reducing adhesion formation in rats using a combination of chitosan and gelatin in different percentages composition and surprisingly observed increased peritoneal inflammation when the percentual of chitosan was higher than 25% [72].

Recently, a novel hydrogel, that is, poly(*ε*-caprolactone)-poly(ethylene glycol)-poly(*ε*-caprolactone) (PCEC) demonstrated potential to prevent postoperative adhesions in rats [73, 74]. PCEC is thermosensitive, and, at body temperature, a solution containing micelles is converted into a hydrogel. The PCEC is biodegradable and had low toxicity *in vitro* and *in vivo* [74]. Gao and Deng [74] evaluated PCEC hydrogel and founded a potential application in the prevention of postoperative adhesions. The hydrogel could adhere to peritoneal wounds and degrade gradually over 7–9 days, transformed into a viscous fluid that was completely absorbed within 12 days [74].

Another potential product is aldehyde dextran associated with *ε*-poly(L-lysine), both present extremely low cytotoxicity. The use of aldehyde dextran associated with *ε*-poly(L-lysine) in rats showed equivalent efficacy to commercial antiadhesion barriers (Seprafilm; Interceed) [75].

Parecoxib administration by different routes (intraperitoneal and intramuscular) significantly decreased the quantity and the severity of abdominal adhesions in rats. In addition, parecoxib administration did not cause healing defects or infectious complications, showing that, in these rat models, parecoxib might reduce adhesions formation and stimulate further investigation in other species [76].

The action of alginate gel in preventing adhesions formation was tested and compared with carboxymethylcellulose membrane [77]. Both compounds presented antiadhesive effectiveness, but no differences were observed between compounds [77].

Although some agents, such as sodium hyaluronate/carboxymethylcellulose (HA/CMC) and oxidized regenerated cellulose, have been approved by the FDA and they are the gold standards for preventing the formation of peritoneal adhesion, they present high cost and difficult handling; much more interest has been directed to using drugs like angiotensin II receptor blockers (ARBs) and HMG-CoA reductase inhibitors (statins) [78]. ARBs decrease TGF-*β* levels and atorvastatin increases the profibrinolytic environment in the peritoneum, which leads to adhesions inhibition [78–80].

Dinarvand et al. (2013) compared the use of losartan (1, 5, and 10 mg/kg), atorvastatin (1, 20, and 30 mg/kg), losartan (10 mg/kg) plus atorvastatin (20 mg/kg), and sodium hyaluronate/carboxymethylcellulose (HA/CMC) administered intraperitoneally in 90 males of mice. After 7 days, the grade of adhesions was scored and the simultaneous intraperitoneal administration of losartan and atorvastatin led to a much higher reduction of adhesions compared with that in the HA/CMC group [78].

### 2.5. Adhesiolysis

When adhesions result in recurrent symptoms, adhesiolysis is recommended. Focal, small, and relatively avascular restrictive adhesions should be sectioned, but extensive and vascular adhesions should be carefully dissected with meticulous attention paid to hemostasis [9, 39].

The prognosis for horses undergoing repeat laparotomy due to adhesions is poor, with reported survival rates of 0–20% [39]. In cases of extensive adhesions formation, the surgical approach can include an incomplete intestinal bypass, leaving the mature obstructive adhesion *in situ*. This technique has the benefit of minimizing inflammation associated with adhesiolysis, which could act as a focus for *de novo* adhesion reformation [9].

Minimally invasive surgery combined with the possibility of viewing areas of the peritoneal cavity not seen in traditional celiotomy makes laparoscopic adhesiolysis a useful modality in equine surgery. Claunch and Mueller [9], Tittel et al. [81], Gutt et al. [82], and Hackethal et al. [83] compared adhesion formation under laparoscopy and laparotomy and observed reductions in adhesion reformation and postoperative complications in laparoscopy.

## 3. Conclusions

Adhesion formation is the most common cause of colic in horses after celiotomy. The prognosis is poor, with survival rates of 0–20% when repeated celiotomy is necessary. Although adhesion formation and inhibition product development are the subjects of several studies, no definite strategy has been developed for horses, and the best option is prophylaxis.

Good surgical techniques combined with anti-inflammatory, antimicrobial and anticoagulant (heparin) treatments, and the mechanical separation of serosal surfaces by intraperitoneal CMC solutions currently demonstrate the best results in horses. Pan-abdominal prophylactic strategies, in contrast with localized strategies, demonstrate superior results in horses because the regions most commonly affected by adhesions are not the regions that experience the initial surgical trauma.

## Abbreviations

ECM: Extracellular matrix
IL-1: Interleukin 1
IL-6: Interleukin 6
TNF-*α*: Tumor necrosis factor
TGF-*β*: Transforming growth factor
tPA: Tissue plasminogen activator
PAI: Inhibitor of tissue plasminogen activator
PGE_2_: Prostaglandin E_2_
PDGF: Platelet derived growth factor
Factor XII: Hageman's factor
Factor 2: Prothrombin
Factor IIa: Thrombin
FDP: Fibrin degradation products
PMN: Polymorphonuclear
EGF: Epidermal growth factor
IGF: Insulin growth factor
FGF: Fibroblast growth factor
VEGF: Vascular endothelial growth factor
CMC: Carboxymethylcellulose
NSAIDs: Nonsteroidal anti-inflammatory drugs
DMSO: Dimethylsulfoxide
rtPA: Recombinant tissue plasminogen activator
NAC: N-Acetyl-cysteine.
